# Multidimensional analysis of anxiety symptoms in patients with chronic obstructive pulmonary disease (COPD)

**DOI:** 10.1038/s41598-025-96007-5

**Published:** 2025-04-02

**Authors:** Li’ao Wang, Dong Miao, Meiying Wang, Gang He, Zhengqiao Li, Yunsheng Hou, Lei Zhang

**Affiliations:** Bethune International Peace Hospital, Shijiazhuang, 050082 Hebei China

**Keywords:** Chronic obstructive pulmonary disease, Anxiety, Latent profile analysis, Risk factors, Nursing, Psychology, Diseases, Medical research, Neurology, Risk factors

## Abstract

To explore the potential classes of anxiety symptoms in patients with chronic obstructive pulmonary disease (COPD) and analyze their distinct characteristics. Convenience sampling was used to select 211 cases of COPD from 12 hospitals in Hebei Province. The following scales were used: General Information Questionnaire, Anxiety Inventory for Respiratory Disease (AIR), BODE index, Montreal Cognitive Assessment (MoCA), and SF-36 Quality of Life scale. Latent profile analysis (LPA) was conducted on the anxiety symptoms of the survey subjects, and univariate analysis and ordinal logistic regression were used to analyze the risk factors of different profiles. Anxiety symptoms among COPD patients were classified into three types: low-risk anxiety type (57.8%), moderate anxiety-fear type (23.2%), and high anxiety-fear type (19.0%). Ordered multinomial logistic regression analysis revealed that the duration of disease, BODE index, MoCA scores, and SF-36 scores were identified as independent risk factors for the potential classes of anxiety symptoms in COPD patients (*p* < 0.05). There is heterogeneity in anxiety symptoms among COPD patients. Medical staff can provide targeted interventions based on the characteristics and risk factors of different populations to alleviate anxiety symptoms.

## Introduction

Chronic obstructive pulmonary disease (COPD) is a chronic inflammatory condition primarily characterized by airflow limitation^1,2^. It is prevalent among smokers and has emerged as a global health concern due to the aging population^3,4^. According to the World Health Organization^5^, COPD currently ranks as the fourth leading cause of death worldwide^6^ and is projected to become the third leading cause of death by 2030^7^. Anxiety is a frequent complication in individuals with COPD and significantly impacts disease progression^8,9^. It manifests as excessive fear and anxiety, accompanied by related behavioral issues that can inflict substantial suffering and harm on individuals, families, and society at large^10^. The prevalence of anxiety in COPD patients varies considerably, ranging from 6 to 70% depending on disease severity and diagnostic tools employed^11^, with reported rates around 44%^12,13^. Existing studies on anxiety in COPD patients typically assess its degree using scoring scales for grouping purposes. However, this approach fails to capture distinct characteristics of anxiety across different groups or account for potential heterogeneity within each group. Latent profile analysis (LPA) offers an alternative method for classifying individuals based on unique symptom patterns. Widely utilized in psychology research^14^, LPA enables the homogeneous grouping of continuous data into subgroups exhibiting similar symptoms. In this study, latent profiling was employed to analyze anxiety symptoms among COPD patients in order to identify subgroups. Furthermore, factors influencing these distinct groups were examined with the aim of providing insights into the heterogeneity of anxiety experienced by individuals with COPD. These findings may inform targeted intervention measures for medical professionals working with such patients.

## Sbujects and methods

### Study sbujects

A total of 211 COPD patients from 12 hospitals in Hebei Province were selected using the convenience sampling method. Inclusion criteria: (1) Meeting the diagnostic criteria for COPD^15^, with a stable condition and imminent discharge. (2) Aged over 40 years old. (3) Absence of significant cognitive impairment. (4) Voluntary willingness to participate in the investigation. Exclusion criteria: (1) Communication barriers. (2) Participation in other research studies. Diagnostic criteria for COPD: Presence of chronic respiratory symptoms caused by abnormal airways and pulmonary vesicles, accompanied by continuous and progressive airflow obstruction; Forced expiratory volume (FEV)/forced vital capacity (FVC) ratio less than 70% within 1 s after bronchodilator inhalation, excluding other diseases. All human participants in this study were informed of the purpose, methods, potential risks, and benefits of the study, and signed a written informed consent form. This study was reviewed and approved by the Ethics Committee of Bethune International Peace Hospital under protocol number 2023-KY-104.

### Methods

#### General information questionnaire

The self-designed questionnaire includes variables such as gender, age, educational attainment, marital status, payment method for medical expenses, per capita monthly income of the household, occupation, current employment status, place of residence, living conditions, smoking status, duration of illness (course of illness), frequency of illnesses in the past year, presence of other chronic diseases, and their specific types. In total, there are 14 variables.

#### Anxiety inventory for respiratory disease (AIR)

Conducted by Professor Yohannes et al.^16^ and the research team of Tianjin Chest Hospital and Tianjin First Central Hospital, the Cronbach’s α coefficient of the scale was 0.92, indicating a high level of internal consistency. The scale consisted of 10 one-dimensional items measured on a Likert scale with scores ranging from 0 to 3 (0 = none; 3 = almost always occurring). The total score ranged from 0 to 30 points, with higher scores indicating more severe anxiety symptoms in patients. In this study, an AIR < 8 indicated a low anxiety level while an AIR > 8 indicated a high level of anxiety^17^. The Cronbach’s α coefficient for the scale in this study was 0.91.

#### Body-Mass index, airflow obstruction, dyspnea, and exercise capacity (BODE) index

The Bartolome R et al. developed a 14-item set of 4 dimensions (body mass index, degree of airflow obstruction, dyspnea, exercise capacity) to evaluate disease severity and prognosis in COPD patients. The 4-level Likert scoring method was employed for assessment. A higher score indicates a poorer prognosis and an increased risk of mortality. The Cronbach’s α coefficient for this scale in this study was calculated to be 0.932.

#### Montreal cognitive assessment (MoCA)

The Cronbach’s α coefficient of the scale, developed by Nasreddine et al. in Canada, was 0.85 and it comprised 11 items for rapid screening of mild cognitive impairment. The total score on this scale is 30 points, with a cutoff of ≥ 26 points indicating normal cognitive function. In this study, the Cronbach’s α coefficient for the scale was 0.834.

#### SF-36 quality of life scale

The health-related quality of life is assessed using the Medical Outcomes Research Group’s scale, which comprises eight dimensions (physiological function, physical pain, general health, vitality, social function, emotional function, and mental health). The total score on this scale ranges from 0 to 100 points and indicates a higher level of health status as the score increases. In this study, the Cronbach’s α coefficient for the scale was 0.812.

### Data collection

The investigators in this study administered questionnaires to patients on the day prior to discharge, providing an explanation of the study’s purpose and requirements. For the elderly or patients with writing impairments, the investigators read and explained the questions before completing them as directed by the patient. Questionnaires were distributed and collected promptly upon completion, with a total of 220 questionnaires distributed and 211 effectively recovered, resulting in an effective recovery rate of 95.9%.

### Statistical methods

The LPA package of Mplus 8.3 software was utilized for conducting potential profile analysis. The LPA model was constructed using the scores of 10 items from the respiratory anxiety scale as explicit variables, while patients’ anxiety symptoms classification served as potential class variables. The fitting indexes for the LPA model primarily included Akaike information criteria (AIC), Bayesian information criteria (BIC), adjusted BIC (aBIC), entropy value, and Bootstrap-based likelihood ratio test (BLRT). Smaller statistical values of AIC, BIC, and aBIC indicate better fit. Entropy ranges from 0 to 1, with closer proximity to 1 indicating more accurate classification. An entropy value of 0.80 suggests that the classification accuracy exceeds 90%, and the Bootstrap-based Likelihood Ratio Test (BLRT) and the Lo-Mendell-Rubin test (LMR) were utilized to compare class differences between k class models and k-1 class models, with a significant p-value suggesting that the k class model significantly outperformed the k-1 class model. Statistical analysis was performed using SPSS 25.0. Measurement data were expressed as $\:\stackrel{-}{x}$±s or M (P_25_, P_75_), while counting data were presented as frequency and percentage. One-way analysis of variance (ANOVA) or rank sum test was used for measurement data, whereas chi-square test or rank-sum test was applied for counting data analyses. Logistic regression analysis was conducted to evaluate the influence of various factors on different classes of anxiety symptoms in COPD patients, with a significance level of α = 0.05.

## Results

### Potential profile analysis of anxiety symptoms in COPD patients

A total of five latent class models were fitted in this study, as shown in Table 1. Among the models, the Class 5 model exhibited the lowest AIC, BIC, and aBIC values. The Class 3 and Class 4 models demonstrated the highest Entropy values, both approaching 1, while the Class 2 model had the lowest Entropy value. The LMRT indicated significance for the Class 2 and Class 3 models, whereas the Class 4 and Class 5 models did not show significance. BLRT demonstrated significance from the Class 2 to the Class 5 models. Previous research has suggested that the proportion of profiles in the overall sample should be at least 5%. Considering all these factors, this study selected the Class 3 model as the optimal solution.

**Table 1:** Fit information for latent class models of anxiety in COPD patients.

Model	AIC	BIC	aBIC	Entropy	LMRT (*p*)	BLRT (*p*)	Class probability
Class 1	5061.715	5128.752	5065.38				
Class 2	3914.941	4018.849	3920.621	0.993	< 0.001	< 0.001	0.5829/ 0.4171
Class 3	3745.911	3886.689	3753.607	0.999	0.0125	< 0.001	0.5782/ 0.2322/ 0.1896
Class 4	3711.1	3888.749	3720.812	0.999	0.5703	< 0.001	0.0142/ 0.2275/ 0.5687/ 0.1896
Class 5	3671.692	3886.21	3683.419	0.994	0.2614	< 0.001	0.4502/ 0.1327/ 0.1469/ 0.1043/ 0.1659

**Fig. 1:**
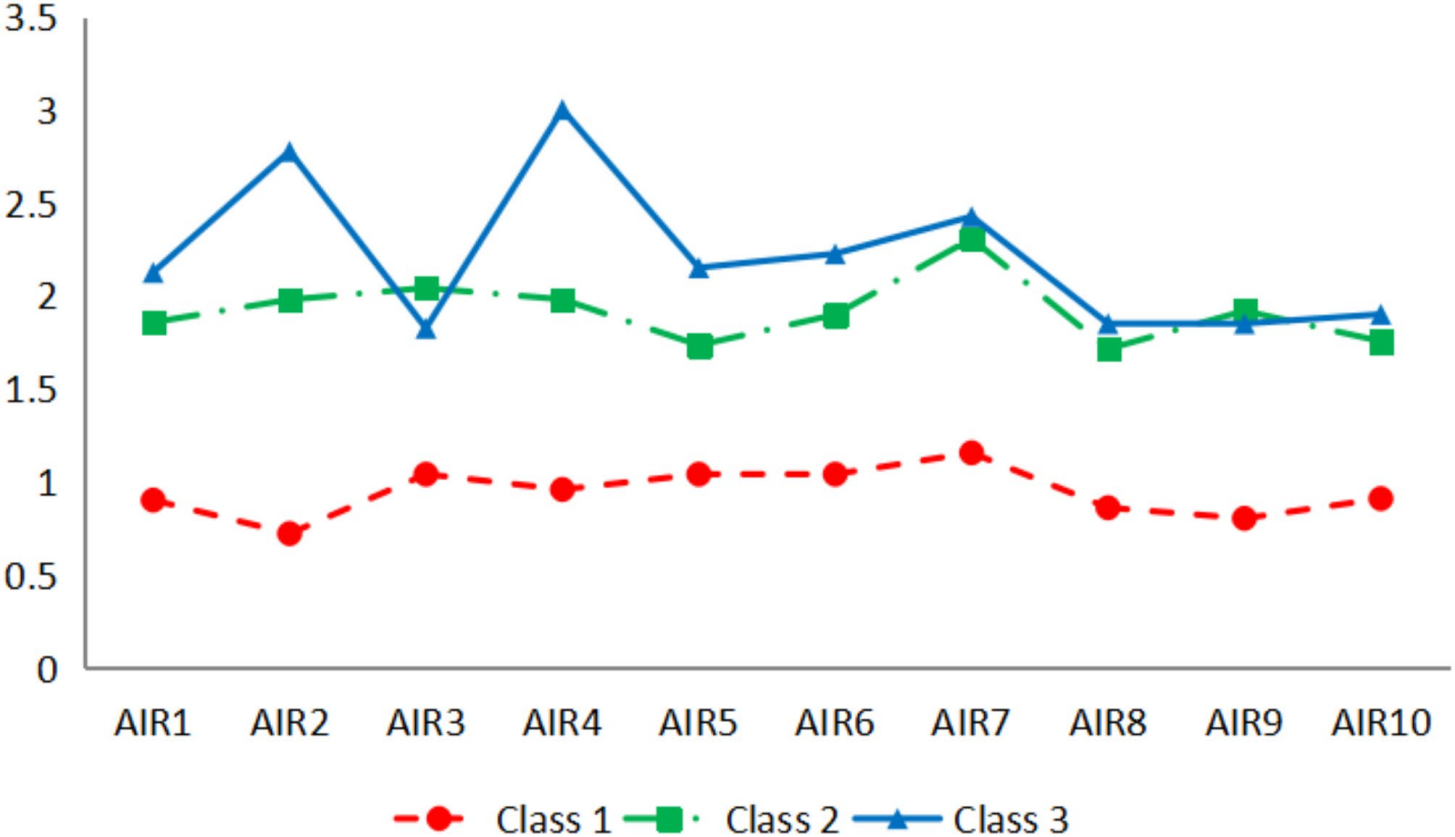
The mean scores of three latent classes on a set of 10 items.

The mean scores of each latent class on the 10 items are depicted in Fig. 1. Class 1 is named “low-risk anxiety type” due to its relatively low scores across all items; it accounts for approximately 57.8% of the samples. Class 3 exhibits significantly higher scores than Class1 on all items with particularly elevated scores on item 2 (I felt very scared or panicked) and item 4 (I had a sense of fear of losing control and/or collapse), which are both related to fear experiences; thus it is labeled as “high anxiety-fear type,” representing around 19% of our samples. The fluctuation pattern observed in Class 2 aligns closely with that seen between Class 1 and Class 3; therefore, it is designated the " moderate anxiety-fear type,” comprising about 23.2% of the samples.

### Univariate analysis of three latent classes of anxiety in COPD patients

There were no significant differences observed in gender, medical payment methods, occupation, employment status, place of residence, living conditions, presence or absence of other chronic diseases, and the frequency of hospitalizations due to COPD exacerbation among the three identified anxiety classes: low-risk anxiety type, high anxiety-fear type, and moderate anxiety-fear type (*p* > 0.05). Conversely, significant differences were noted in nine independent variables, including age, education level, marital status, per capita family monthly income, smoking status, duration of illness, BODE index, MoCA scores, and SF-36 scores (*p* < 0.05), as detailed in Table 2.

**Table 2:** Comparison of demographic characteristics among three latent classes of anxiety symptoms in COPD patients.

Project	Low-risk anxiety type	Moderate anxiety-fear type	High anxiety-fear type	Statistics	*P*
	(*n* = 122)	(*n* = 49)	(*n* = 40)		
Gender				χ2 = 0.68	0.712
Male	76(62.30)	30(61.22)	22(55.00)		
Female	46(37.70)	19(38.78)	18(45.00)		
Age(Years)				χ2 = 16.692	0.010*
40–50	5(4.10)	10(20.41)	6(15.00)		
50–60	5(4.10)	15(30.61)	15(37.50)		
60–70	38(31.15)	12(24.49)	14(35.00)		
>70	39(31.97)	12(24.49)	5(12.50)		
Level of education				χ2 = 13.801	0.032*
Primary school and below	52(42.62)	17(34.69)	11(27.50)		
Junior high school	36(29.51)	13(26.53)	8(20.00)		
High school or technical secondary school	24(19.67)	9(18.37)	9(22.50)		
College or above	10(8.20)	10(20.41)	12(30.00)		
Marital status				χ2 = 6.26	0.044*
Married	103(84.43)	33(67.35)	31(77.50)		
Single	19(15.57)	16(32.65)	9(22.50)		
Payment methods for medical expenses				χ2 = 5.167	0.271
Public expense	15(12.30)	11(22.45)	4(10.00)		
Medical insurance	95(77.87)	31(63.27)	30(75.00)		
Self-paying	12(9.84)	7(14.29)	6(15.00)		
Profession				χ2 = 9.098	0.825
Worker	23(18.85)	15(30.61)	9(22.50)		
Farmer	25(20.49)	8(16.33)	10(25.00)		
Administrative personnel	9(7.38)	4(8.16)	3(7.50)		
Professionals	14(11.48)	2(4.08)	5(12.50)		
Company employee	21(17.21)	10(20.41)	4(10.00)		
Commercial staff	14(11.48)	7(14.29)	5(12.50)		
Service worker	7(5.74)	1(2.04)	1(2.50)		
Others	9(7.38)	2(4.08)	3(7.50)		
On-job				χ2 = 4.132	0.127
Yes	34(27.87)	17(34.69)	18(45.00)		
No	88(72.13)	32(65.31)	22(55.00)		
Residence				χ2 = 3.273	0.195
Rural areas	66(54.10)	34(69.39)	28(70.00)		
Urban areas	56(45.90)	15(30.61)	12(30.00)		
Per capita monthly household income(yuan)				χ2 = 15.801	0.015*
<2000	47(38.52)	33(67.35)	22(55.00)		
2000–4000	31(25.41)	9(18.37)	7(17.50)		
4001–6000	29(23.77)	2(4.08)	8(20.00)		
>6000	15(12.30)	5(10.20)	3(7.50)		
Status of residence				χ2 = 3.273	0.195
Live alone	34(27.87)	15(30.61)	6(15.00)		
Not live alone	88(72.13)	34(69.39)	34(85.00)		
Smoking status				χ2 = 13.219	0.010*
Smoke	15(12.30)	1(2.04)	1(2.50)		
Used to smoke, now quit smoking	33(27.05)	9(18.37)	5(12.50)		
Don’t smoke	74(60.66)	39(79.59)	34(85.00)		
Duration of illness (Year)				χ2 = 24.791	0.000 ***
1–2	25(20.49)	8(16.33)	4(10.00)		
2–4	28(22.95)	10(20.41)	2(5.00)		
5–10	52(42.62)	20(40.82)	14(35.00)		
>10	17(13.93)	11(22.45)	20(50.00)		
Times of hospitalizations due to COPD exacerbations in the past year				χ2 = 6.21	0.4
≤ 2	33(27.05)	14(28.57)	13(32.50)		
3	22(18.03)	14(28.57)	12(30.00)		
4	40(32.79)	12(24.49)	7(17.50)		
≥ 5	27(22.13)	9(18.37)	8(20.00)		
Combined with other chronic diseases				χ2 = 2.358	0.308
Yes	97(79.51)	39(79.59)	36(90.00)		
No	25(20.49)	10(20.41)	4(10.00)		
BODE index	5.28 ± 2.92	7.10 ± 2.07	7.60 ± 1.79	*F* = 16.827	0.000 ***
MoCA scores	21.60 ± 5.25	18.47 ± 5.97	16.25 ± 5.54	*F* = 16.393	0.000 ***
SF-36 scores	65.96 ± 12.55	62.71 ± 10.86	54.38 ± 10.45	*F* = 14.525	0.000 ***

*p < 0.05 ** *p* < 0.01 *** *p* < 0.001.

### Logistic regression analysis of anxiety symptoms in COPD patients

The anxiety classes of COPD patients were designated as the dependent variables, while the nine statistically significant variables mentioned above served as independent variables, with specific assignments detailed in Table 3. Logistic regression analyses were conducted twice, using the low-risk anxiety type, high anxiety-fear type, and moderate anxiety-fear type as reference groups; the results are presented in Tables 4 and 5.

**Table 3:** Variable assignment.

Variable	Assignment
Age (years)	1 = 40–50; 2 = 50–60; 3 = 60–70; 4=>70
Education	1 = Primary and below; 2 = Junior high school; 3 = High school or junior college; 4 = Bachelor degree or above
Marital status	1 = Married; 2 = Unmarried
Per capita monthly household income (yuan)	1=<2000; 2 = 2000–4000; 3 = 4001–6000; 4=>6000
Smoking status	1 = Smoking; 2 = Used to smoke, now quit; 3 = No smoking
Duration of illness (years)	1 = 1–2; 2 = 2–4; 3 = 5–10; 4=>10
BODE index	Original value entry
MoCA scores	Original value entry
SF-36 scores	Original value entry

**Table 4:** Logistic regression of latent classes of anxiety in COPD patients (The low-risk anxiety group was utilized as the reference group, *n* = 211).

Variable	Moderate anxiety-fear type VS Low-risk anxiety type				High anxiety-fear type VS Low-risk anxiety type			
	β	Waldχ2	*p*	OR(95%CI)	β	Waldχ2	*p*	OR(95%CI)
Age (years)	− 0.356	2.866	0.09	0.7 (0.463 ~ 1.058)	-0.449	3.233	0.072	0.638 (0.391 ~ 1.041)
Education	0.024	0.016	0.898	1.025 (0.705 ~ 1.489)	0.226	0.973	0.324	1.254 (0.800 ~ 1.965)
Marital status	0.867	3.626	0.057	2.379 (0.975 ~ 5.805)	0.389	0.424	0.515	1.476 (0.457 ~ 4.761)
Per capita monthly household income (yuan)	− 0.435	4.146	0.042	0.647 (0.426 ~ 0.984)	-0.193	0.655	0.419	0.825 (0.517 ~ 1.316)
Smoking status	0.767	4.302	0.038	2.154 (1.043 ~ 4.446)	0.783	2.551	0.11	2.189 (0.837 ~ 5.724)
Duration of illness (years)	0.07	0.124	0.725	1.072 (0.728 ~ 1.580)	0.746	7.712	0.005	2.109 (1.245 ~ 3.570)
BODE index	0.422	15.308	0	1.343 (1.234 ~ 1.884)	0.422	15.308	0	1.525 (1.234 ~ 1.884)
MoCA scores	− 0.146	9.912	0.014	0.917 (0.789 ~ 0.946)	-0.146	9.912	0.002	0.864 (0.789 ~ 0.946)
SF-36 scores	− 0.092	16.727	0.139	0.976 (0.872 ~ 0.953)	-0.092	16.727	0	0.912 (0.872 ~ 0.953)

Nagelkerke R^2^ = 0.506.

**Table 5:** Logistic regression of latent classes of anxiety in COPD patients (The moderate anxiety-fear type was utilized as the reference group, *n* = 211).

Variable	High anxiety-fear type VS Moderate anxiety-fear type			
	β	Waldχ2	*p*	OR(95%CI)
Age (years)	− 0.045	0.031	0.859	0.956 (0.582 ~ 1.570)
Education	0.161	0.543	0.461	1.175 (0.765 ~ 1.804)
Marital status	− 0.046	0.006	0.938	0.955 (0.298 ~ 3.063)
Per capita monthly household income (yuan)	0.072	0.075	0.784	1.074 (0.643 ~ 1.796)
Smoking status	0.475	0.698	0.404	1.608 (0.527 ~ 4.907)
Duration of illness (years)	0.593	4.825	0.028	1.809 (1.066 ~ 3.070)
BODE index	0.07	0.239	0.625	1.072 (0.811 ~ 1.418)
MoCA scores	− 0.049	1.088	0.297	0.953 (0.869 ~ 1.044)
SF-36 scores	− 0.078	8.888	0.003	0.925 (0.879 ~ 0.974)

Nagelkerke R^2^ = 0.307.

#### Logistic regression analysis was conducted with the low-risk anxiety type as the reference group

Table 4 illustrates that, in comparison to the low-risk anxiety type, the factors such as family per capita monthly income (OR = 0.647, *P* = 0.04), smoking status (OR = 2.154, *P* = 0.038), BODE index (OR = 1.34, *P* < 0.01), and MoCA scores (OR = 0.917, *P* < 0.01) were associated with a lower likelihood of being classified as the moderate anxiety-fear type; conversely, a longer duration of illness (OR = 2.109, *P* = 0.005), higher BODE index (OR = 1.525, *P* < 0.01), lower MoCA scores (OR = 0.864, *P* = 0.014), and lower SF-36 scores (OR = 0.912, *P* < 0.01) increased the likelihood of classification as the high anxiety-fear type.

#### Logistic regression analysis was carried out with the moderate anxiety-fear type as the reference group

According to Table 5, individuals with a longer duration of illness (OR = 1.809, *P* = 0.028) and lower SF-36 scores (OR = 0.925, *P* = 0.003) were more likely to be classified as having high levels of anxiety and fear compared to those with moderate levels. In conclusion, patients with a longer duration of illness (OR > 1) and lower SF-36 scores (OR < 1) exhibited a higher likelihood of being classified as the high anxiety-fear type compared to those with low or moderate anxiety-fear types. Low family monthly income per capita and smoking were more likely to be classified as the moderate anxiety-fear type when considering the low-risk anxiety type as the reference group. Furthermore, a higher BODE index and lower MoCA scores were associated with an increased probability of being classified as both the moderate anxiety-fear type and high anxiety-fear type. Patients with a longer duration of illness and lower SF-36 scores had an elevated likelihood of being classified as the high anxiety-fear type. When using the moderate anxiety-fear type as the reference group, patients with a prolonged duration of illness and low SF-36 scores demonstrated an increased tendency to be classified as the high anxiety-fear type.

## Discussion

### Anxiety symptoms in COPD patients were classified into three latent classes

This study revealed heterogeneity in anxiety among COPD patients, encompassing three distinct types: the low-risk anxiety type (57.8%), the moderate anxiety-fear type (23.2%), and the high anxiety-fear type (19.0%). Notably, the combined proportion of moderate and high anxiety-fear types reached 42.2%, which aligns with findings from relevant foreign studies^18–20^. This can be explained by several factors. Firstly, the chronic nature of the disease, physical limitations, social isolation, physiological impact on the brain caused by COPD, and genetic factors contribute to patients experiencing low mood and diminished self-efficacy, ultimately leading to symptoms of anxiety^21^. Secondly, there is an overlap between anxiety symptoms and physical manifestations in COPD patients^22–23^. For instance, acute episodes of fear or panic can manifest as sudden shortness of breath that exacerbates dyspnea development either acutely or chronically. Chronic anxiety presents as an overwhelming feeling of fear or apprehension that hinders the activities of daily life and limits the participation in social interactions; furthermore, these anxiety symptoms often trigger or worsen COPD symptoms^24–26^. Due to this intricate interplay between various factors, the clinical manifestations of anxiety differ significantly among COPD patients. Some studies^27–28^ have classified levels of anxiety in COPD patients into mild, moderate, and those without any signs of clinical-level anxieties based on their scores on a clinical scale for measuring anxiety, and these findings align with the results obtained in this study, however, the distinction lies in this study’s more precise classification and identification of anxiety categories in patients with COPD. This allows nursing staff members to promptly recognize different types of anxieties present among COPD patients and develop targeted intervention plans accordingly, in order to maximize the effectiveness.

### Analysis of risk factors of potential classes of anxiety symptoms in COPD patients

#### Patients with a low duration of illness and low SF-36 scores are more likely to be classified as “high anxiety-fear type"

Compared to the patients classified as “low-risk anxiety type” and “moderate anxiety-fear type,” those with a prolonged duration of illness (OR > 1) and lower scores on the quality of life questionnaire SF-36 scores (OR < 1) were more likely to be classified as the high anxiety-fear type. It is suggested that the patients with a longer disease duration and lower quality of life scores are more prone to experiencing heightened levels of anxiety and fear. Research has indicated that the duration of illness and quality of life in COPD patients serve as predictors of anxiety, potentially due to such factors as increased hospitalizations, worsening conditions^29–31^, elevated medical expenses, impaired self-care abilities resulting from the disease, associated disabilities^32^, distorted self-image, diminished quality of life, physical and mental dysfunction, as well as fear of social interactions^33^. Studies have also demonstrated a correlation between the severity of COPD symptoms and anxiety levels among patients; as the disease progresses, so does their level of anxiety. Furthermore, research^34^ has shown that anxiety negatively impacts the overall quality of life while simultaneously being influenced by it. The presence of dyspnea, reduced exercise tolerance, and limited daily activities caused by COPD significantly affect patients’ quality of life, leading to an exacerbation of their level of anxiety. These findings align with the results obtained from this study. Therefore, clinical staff should pay more attention to the quality of life of patients while treating physical diseases. Specific intervention measures include: (1) Physiological management: optimizing patient breathing training through pursed lip breathing and abdominal breathing, promoting lung rehabilitation through personalized exercise training such as walking, cycling, etc., and providing standardized medication and oxygen therapy support guidance to patients; (2) In terms of psychological intervention, patients’ anxiety symptoms are alleviated through cognitive-behavioral therapy, mindfulness and relaxation training, emotional support, and empathetic communication; (3) Encouraging family participation in nursing and community service utilization to reduce the burden on patients’ lives; (4) Regularly conducting psychological screening for patients and adjust treatment plans.

#### Low family monthly income per capita and smoking were more likely to be classified as moderate anxiety-fear type

Compared to the low-risk anxiety type, individuals with lower per capita family income (OR = 0.647, *P* = 0.04) and those who smoke (OR = 2.154, *P* = 0.038) were more likely to be classified as having moderate anxie-fear symptoms. This could be attributed to the fact that COPD is a chronic disease with a prolonged course. The high disability rate and frequent hospitalizations associated with COPD lead to increased medical costs, which further exacerbate the economic burden on patients and contribute to their anxiety levels. Xu Yinfang et al.‘s findings also support this notion by highlighting how low income and heavy financial burdens intensify patients’ anxiety levels, aligning with similar results from foreign studies^35–37^ as well as this study. Smoking is a primary etiological factor in COPD. Numerous international studies have demonstrated that the duration of smoking, severity of nicotine dependence, heightened anxiety levels, and younger age contribute significantly to the perpetuation of smoking among COPD patients^38^. Furthermore, research has established a correlation between smoking and alterations in neurotransmitter systems, inflammation, and increased symptoms or disorders related to oxidative stress-induced anxiety^39^. Smoking exacerbates dyspnea symptoms in patients with COPD, leading to disease exacerbation and heightened anxiety levels^40,41^. As dyspnea worsens and physical activity declines in individuals with COPD, they experience an escalation in anxiety symptoms, which further drives their tobacco consumption, resulting in a deteriorated quality of life^42^. Additionally, there exists an interplay between anxiety symptoms and dyspnea along with chest tightness—findings consistent with those observed in this study. Smoking cessation serves as the cornerstone for mitigating lung function decline while reducing hospitalization rates and enhancing survival outcomes. Therefore, it is imperative for healthcare professionals to provide comprehensive understanding and support to patients during their smoking cessation journey by fostering confidence-building strategies aimed at alleviating associated anxiety. Specific intervention measures include: firstly, providing certain economic support and resource integration for low-income patients, such as government medical subsidies, funding from charitable organizations, and promoting community health services to reduce the economic burden on patients; secondly, assisting patients in quitting smoking through behavioral interventions, alternative therapies, psychological counseling, and other methods.

#### Higher BODE index and lower MoCA scores are more likely to be classified as the moderate anxiety-fear type and high anxiety-fear type

Compared to the low-risk anxiety type, individuals with a higher BODE index (OR = 1.34, *P* < 0.01) and lower MoCA scores (OR = 0.917, *P* = 0.014) were more likely to be classified as having the moderate-fear anxiety type. Those with a high BODE index (OR = 1.525, *P* < 0.01) and low MoCA scores (OR = 0.864, *P* = 0.014) were more likely to be classified as having the high anxiety-fear type. There was no significant difference in the BODE index and MoCA scores between the “moderate anxiety-fear type” and “high anxiety-fear type”. The higher the BODE index and the lower the MoCA scores, the more severe the symptoms of anxiety become. Specific intervention measures include: firstly, guiding patients to use respiratory techniques and reasonable oxygen therapy to alleviate respiratory distress symptoms, and reducing patients’ BODE scores through exercise rehabilitation and nutritional support; secondly, improving patients’ cognitive level through cognitive training, simplified health information, and family support; thirdly, providing psychological and social support, regularly following up with patients through phone or mobile medical platforms, monitoring their anxiety symptoms and changes in their condition; fourthly, evaluating on a regular basis the BODE and MoCA scores of patients and adjusting intervention measures in a timely manner.

## Summary

In this study, the LPA method was utilized to stratify anxiety symptoms in COPD patients, facilitating more accurate identification and analysis of patient anxiety classifications by clinical nurses. This enables the formulation of personalized nursing plans and offers novel perspectives for precision nursing. However, there are certain limitations to this study: (1) It is an observational cross-sectional study with limited causal inference power. (2) This study adopted convenience sampling method to include the research subjects, which has potential selectivity bias. The main reason is that the research subjects are selected from specific medical institutions and the patients with mild conditions, home management, and those from rural areas were not included, resulting in insufficient sample representativeness. Moreover, patients who voluntarily participated in the study may have higher health awareness and pay more attention to their own anxiety issues, leading to the potential bias in self selection effect. (3) The research included COPD patients from Hebei Province alone, thus limiting the generalizability of the findings to COPD patients nationwide.

## Data Availability

The data supporting the results of this study can be provided upon request to the corresponding author. Due to privacy or ethical restrictions, these data are not publicly available.
